# The Pre-implementation Process of Adapting a Culturally Informed Stress Reduction Intervention for Native American Head Start Teachers

**DOI:** 10.1007/s43477-022-00070-3

**Published:** 2023-01-09

**Authors:** Deborah H. Wilson, Katie E. Nelson, Ashley Gresh, Adriann Ricker, Shea Littlepage, Lydia Koh Krienke, Teresa N. Brockie

**Affiliations:** 1 School of Nursing, Johns Hopkins University, 525 N Wolfe St., Baltimore, MD 21205 USA; 2 Johns Hopkins Bloomberg School of Public Health, Center for Indigenous Health, 415 N. Washington St. 4th Floor, Baltimore, MD 21231 USA

**Keywords:** Community-based participatory research, Native American, Process evaluation, Cultural safety

## Abstract

Head Start is a federally funded program for children (3–5 years) from low-income families. In the Fort Peck Native American Reservation, tribal Head Start teachers have reported high stress in supporting children experiencing adverse childhood experiences. Thus, we adapted the *Little Holy One* intervention (ClinicalTrials.gov: NCT04201184) for the teachers’ context and culture to enhance psychological health and well-being. Within a participatory framework, the eight-step ADAPT-ITT methodology was used to guide the adaptation process: assessment; decision; adaptation; production; topical experts; integration; training; and testing. For Step 1, we purposive sampled 27 teachers, ancillary staff, and parents to understand teachers’ stress, support mechanisms, and interest in an intervention via focus groups (*n* = 9) and individual interviews (*n* = 18). Qualitative data underscored teachers’ experiences of stress, depression, and need for support (Step 1). Iterative feedback from a tribal advisory board and *Little Holy One* designers rendered selection of five lessons (Step 2, 5), which were adapted for the teachers via theater testing (Step 3, 4). Community capacity assessment revealed their ability to implement the intervention (Step 6). Testing of this adapted intervention in a feasibility trial (steps 7, 8) will be reported in a future publication. A rigorous systematic process within a participatory framework allowed intervention adaption based on community input. Leveraging “culture as treatment” may be useful for enhancing psychological health outcomes for Native Americans who historically underutilize existing psychological services.

**Supplementary Information:**

The online version contains supplementary material available at 10.1007/s43477-022-00070-3.

## Background

### Adverse Childhood Experiences

Native Americans residing in remote reservations benefit from connection to place, culture, community, and tribal sovereignty (Gone & Trimble, 2012). However, many Native Americans still struggle with poverty, high crime rates, poorly performing schools, and lack of healthcare services and job opportunities (Brockie et al., 2013; Gone & Trimble, 2012; Wexler et al., 2015). The prevalence of adverse childhood experiences (ACEs)—stressful or traumatic events in early life that can last into adulthood and contribute to increased morbidity and mortality across the lifespan (Felitti et al., 1998)—compounds less-than-optimal social health determinants.

National surveillance data shows ACE scores among Native Americans to be 2.36 times higher than those of white individuals (Giano et al., 2020). Furthermore, Native American children who are exposed to or witness traumatic events, such as intimate partner violence and/or physical and sexual abuse and neglect, experience post-traumatic stress disorder (PTSD) twice as much as the general United States (U.S.) population (Brockie et al., 2015). A prior study with Fort Peck youth ages 15–24 found that 78% of the sample experienced at least one ACE (Brockie et al., 2015). After controlling for demographics, each additional ACE increased the odds of PTSD by 55%, depression by 57%, suicide attempts by 37%, and high-risk substance use by 51% (Brockie et al., 2015). Culturally specific ACEs, i.e., higher levels of discrimination and historical loss, were significantly related to multiple risk behaviors and mental health outcomes, although not suicide attempts. Other studies have found positive, statistically significant associations between suicide attempts and familial attendance at residential boarding schools (Hackett et al., 2016; McQuaid et al., 2017), but not in the context of an ACE study.

Prevalence of ACEs varies by region, primarily due to the impact of social determinants of health (e.g., poverty, access to healthcare, healthy foods, crime rates, and life expectancy); however, the overall picture for Native Americans is alarming. Substantive evidence suggests that higher rates of ACEs among Native American populations can be attributed to historical traumas experienced as a direct result of colonization (e.g., the outlawing of language and culture, forced relocation to reservations, and child abuse in the boarding school system) (Brockie et al., 2015; Giano et al., 2020; Gone & Trimble, 2012). Recent traumas stemming from ongoing discrimination have also had an effect. These factors have spurred intergenerational trauma, the experience of severe traumas transmitted to offspring through epigenetic changes affecting children’s physical and psychological health and well-being for generations (Brockie et al., 2013; Gone, 2013; Yehuda & Lehrner, 2018). Factors that can help children with ACEs to overcome adversity include education and positive childhood experiences (Bethell et al., 2019). Head Start teachers provide safe, stable, and nurturing relationships that can protect against mental health challenges and deter risk behaviors as children grow into adulthood (Baum et al., 2018; Bethell et al., 2019).

### Head Start

Head Start is the largest federally funded national early-childhood education program in the U.S. (Administration for Children and Families, [ACF], 2019)*.* Designed to help break the cycle of poverty, Head Start provides preschool children ages 3–5 from low-income families with a comprehensive program that meets their emotional, social, health, nutritional, and psychological needs (ACF, 2019). Head Start tends to some of the most underserved, marginalized communities and vulnerable children in the U.S. (Harding et al., 2019). The program is divided into 12 regions, 10 of which are geographically based. The populations served define the other two: Region XI covers programs operated by federally recognized Native American/Alaska Native tribes, and Region XII serves migrant and seasonal workers and their families (ACF, 2019).

#### Tribal Head Start Teacher Stress

Despite Head Start teachers reporting a high level of job satisfaction, especially in their ability to make a difference in their student’s lives (Bullough & Hall-Kenyon, 2011; Gibson, 2013), early childhood education teaching remains one of the most stressful occupations in the U.S., with 46% of teachers reporting daily stress during the school year (Carroll, 2007). Teacher stress can lead to burnout, anxiety, depression, sleep disorders, poor performance, and a high turnover rate. Secondary traumatic stress, or compassion fatigue, also contributes to teachers’ strain as they work to support children and families that have suffered multiple adverse experiences (Hydon et al., 2015; Sharp Donahoo et al., 2018). In poverty-stricken areas such as Native American reservations, Head Start teachers are likely to come from the same community as their students and thus deal with similar ACEs and stressors as the children they teach. These ACEs can influence the teachers’ stress levels and hinder their well-being (Hughes et al., 2017).

Head Start teachers, particularly those working in rural, low-income communities, are well-positioned to help mitigate ACEs’ negative and/or long-term effects. Teachers can provide positive interactions and emotional and instructional support, are sensitive and responsive, and stimulate children’s development. They are often trusted, can effectively communicate, develop good relationships with parents, and often serve as first responders for students in times of crisis (Baum et al., 2018). As such, expanding teachers’ resilience skills—providing them with tools to cope with their stress and trauma—can potentially have a lasting impact on their well-being as well as that of the students, schools, and communities (Baum et al., 2018; Jeon et al., 2014).

### Reservation Community Needs Assessment

In rural northeastern Montana, the Fort Peck Reservation, home to the Assiniboine and Sioux tribes, has experienced extreme health inequities and collective traumas. Fort Peck’s tribal Head Start administration reports that teachers need enhanced support, given that they work in a challenging environment with a population struggling with heightened stress, all while receiving minimal assistance for managing their own health and well-being. A community health assessment conducted at Fort Peck in 2015 showed that adults in Fort Peck averaged 4.4 mental health days compared to 3.4 among other Montana residents. This need is compounded by the limited number of mental health providers (1 to 1030 Fort Peck reservation residents) as compared across with the rest of Montana (1 per 399 residents). Differences in the ratio of primary healthcare providers per residents is even more striking (1 provider to 5563 in the Fort Peck Reservation versus 1 to 1312, in the rest of the state of Montana (Fort Peck Tribal Health Department, 2016).

There is a critical need for sustainable psychological and behavioral health support in Fort Peck (Brockie et al., 2021). However, wait times of up to three months, a lack of private or employment-based health insurance, and the remote location of reservations hampers service provisions (Gone & Trimble, 2012; Tracey, 2007). Evidence also suggests that Native Americans are reluctant to utilize existing health services because of structural and institutional racism (Gone & Trimble, 2012; Tracey, 2007). Furthermore, the Indian Health Service, the primary healthcare provider on reservations, is chronically underfunded and understaffed (Siddons, 2018). As a result, Fort Peck, like many reservation communities, lacks access to quality, culturally sensitive psychological and behavioral healthcare (Wexler & Gone, 2012).

### Cultural and Community Strengths

Despite ongoing disparities, Native American communities demonstrate immense cultural and community strengths. The psychological health challenges afflicting Native American communities have been connected to settler colonialism, a system of oppression based on colonization and genocide to displace an indigenous population and replace it with a new settler population (Brave Heart, 2003; Brockie et al., 2013; Gone, 2007; McCarthy & Cornell Law School, 2022). This awareness has led to an emergence of behavioral health interventions that emphasize cultural buffers (e.g., positive tribal identity and communal mastery) to promote well-being and bolster individual and collective strengths through connection to cultural heritage (Gone & Alcántara, 2007; Walls et al., 2016; Wexler, 2013). Furthermore, culturally informed psychological healthcare and even “culture as treatment,” defined as the inclusion of traditional healing practices, cultural values, and messaging to replace mainstream models of health and policy intervention (Green, 2010), holds the promise of carrying forward cultural strengths for generations to come (Gone & Trimble, 2012; O'Keefe et al., 2019).

## Study Purpose

The anecdotal experiences of our colleagues in Fort Peck, paired with limited existing literature, clearly point to a gap in implementation research that supports the psychological health and well-being of tribal Head Start teachers. Therefore, the purpose of this paper is to:

1. Describe our study methodology using the principles of Community-Based Participatory Research (CBPR), and ADAPT-ITT methodology within an existing academic-community partnership,
2. Outline the process of finding, adapting, and preparing an intervention for testing that will support tribal Head Start teachers by reducing stress and depression, improving well-being, and strengthening resilience.

### Literature Review

First, the research team conducted a literature review to comprehend the landscape of resilience-based interventions implemented or culturally adapted for Head Start teachers across the U.S. (Wilson et al., 2022). The search highlighted what potential options, if any, existed before engaging with community partners and determining which intervention would best meet the needs of tribal Head Start teachers.

The literature search found the following successful evidence-based resources implemented to support Head Start teachers in coping with the demands of their profession: mindfulness-based cognitive therapy (Gold et al., 2010); access to mental health consultants and social workers (Raver et al., 2008) and self-care and classroom-management skills (Rombaoa Tanaka et al., 2020). We did not find any studies that centered on or prioritized Native American culture, values, and strengths (such as tribal and/or spiritual identity) or challenges (such as historical trauma, discrimination, and health disparities) when adapting and implementing psychological health and wellness interventions. These findings motivated the shift from adapting an existing evidence-based psychological intervention to exploring and choosing Wakȟáŋyeža, the culturally informed intervention hereafter referred to by its translation, *Little Holy One.*

#### Little Holy One

Based on essential preliminary research and feedback, we opted to adapt an intervention currently used on the Fort Peck Reservation for Head Start parent–child dyads (ClinicalTrials.gov: NCT04201184). The randomized controlled trial (RCT), *Little Holy One*, is an intergenerational intervention designed to reduce parental stress and trauma-related symptoms among parent–child dyads. The children are ages 3–5 and attend tribal Head Start (Brockie et al., 2021). The intervention is a strengths-based, 12-module curriculum focused on promoting family wellness across a holistic well-being (i.e., physical [behavioral], emotional, mental [cognitive], and spiritual) spectrum. There are four cultural components in the *Little Holy One* curriculum designed to support the psychological health and well-being of parent–child dyads by:

1. bolstering tribal identity.
2. working on communal mastery (group efficacy).
3. addressing contemporary and historical trauma.
4. promoting smudging (a traditional way to remove negative thoughts, feelings, and foster positive energy by burning herbs such as sage or cedar to create smoke that cleanses the air and those within it (Charleyboy, 2012).

With support from the principal investigator of *Little Holy One*, our tribal advisory board, and the Head Start administration, we adapted the four cultural lessons into a stress-reduction curriculum for Head Start teachers. Community members and a Fort Peck-based tribal advisory board initially developed the cultural lessons with pilot data demonstrating that lessons were feasible and acceptable to participants. Table 1 outlines the lessons taken from the *Little Holy One* curriculum for adaptation to the tribal Head Start teacher context.

**Table 1:** Lesson description

Lessons for adaptation in original order	Description of lessons	Lesson Activities and time to complete
Promoting tribal identity cultural lesson	Connects one to the creator, responsibility to live a good life by walking spiritual path	Practice greeting of relatives in Nakoda and Dakota Traditional naming 1 h
Understanding our Emotions^a^ CETA lesson	Understand association among thoughts, feelings, and behavior	Identifying depression, managing anger and stress; working through challenges Visualization activity 1 h
Smudging cultural lesson	Therapeutic healing practice to resolve unsettling feelings and thoughts	Smudging together; smudging as a daily routine 45 min
Strengthening family and community cultural lesson	Therapeutic value of connectedness to relatives and community	Knowing our relatives family tree exercise. My friends and family exercise 1 h
Healing historical and contemporary trauma cultural lesson	Identify imbalances in physical, emotional, mental, and spiritual domains created by historical trauma	Identifying and coping with effect of historical traumas. Strength and resilience Forgiveness exercise Smudge at end 1 h

^a^This lesson was added during the adaptation process

### Ethics Approval

The Fort Peck Head Start administration expressed interest in exploring and implementing a sustainable intervention to promote their teachers’ well-being, which was solidified in a meeting with the Tribal Council, consisting of Assiniboine and Sioux tribal leaders. We discussed our intent to adapt *Little Holy One* to the tribal Head Start teacher context. A Tribal Resolution (#30-348-2020-03) authorized this research, permitting the team to apply for funding and conduct a study on the reservation with Fort Peck Head Start teachers. Additionally, the resolution includes a section on the tribe’s rights to protect their intellectual property and Indigenous knowledge (e.g., pictures, songs, and stories). It also requires that the council review any manuscripts before publication. XXXX University (Blinded for review) and the Fort Peck Tribal Institutional Review Board (IRB) granted IRB approval.

## Research Design and Setting

### Study Design

This study uses qualitative data from observation, interviews, and focus groups to adapt a preventative psychological health intervention using the ADAPT-ITT process within a Community-Based Participatory Research (CBPR) framework for Native American Head Start teachers on the Fort Peck Reservation. The Template for Intervention Description and Replication (TIDieR) checklist (Hoffmann et al., 2014) notes the adaptation process (Additional File 1). The Consolidated Criteria for Reporting Qualitative Research (COREQ) (Tong et al., 2007) describes the interviewing and focus-group process (Additional File 2).

### Study Setting and Population

Spanning 180 miles and encompassing 3200 square miles, the Fort Peck Reservation is home to approximately 12,000 members of the Assiniboine and Sioux tribes (Fort Peck Assiniboine & Sioux Tribes, 2022). Six Head Start schools across the Fort Peck Reservation serve over 300 children ages 3–5, pregnant women, and children from birth to age 3 and children and families who are homeless, in foster care, or receiving Temporary Assistance for Native Families or Supplemental Security Income (Fort Peck Assiniboine & Sioux Tribes, 2022). The Fort Peck Tribal Head Start administration reports that most children come from single-parent homes, and 80% live below the federal poverty level (Personal communication V. Wood, July 10, 2020).

There are 21 Head Start lead teachers and teacher’s assistants in Fort Peck. All identify as female and Native American, primarily Assiniboine or Sioux; more than half have worked for Head Start for over 20 years. To protect participant identity, specific demographic data is not reported. Fort Peck Head Start administration also reports that teachers receive no mental health support, despite the stresses of their job, personal life, and community such as, drug addiction, high crime, and suicide rates (Personal communication V. Wood, July 10, 2020). Quantitative data that reports explicitly on the Fort Peck Head Start teachers’ mental health is not publicly available. However, the American Indian and Alaska Native Family and Child Experiences Study which encompasses a nationally representative sample, underscores that 19% of Native American Head Start children’s lead teachers report mild depression, 11% report moderate depression, and 5% report severe depression (American Indian and Alaska Native Head Start Family and Child Experiences Survey, 2019).

Existing services on the reservation include two Substance Abuse and Mental Health Services Administration (SAMHSA)–funded initiatives: *Native Connections*, which works to reduce substance abuse and suicide in Fort Peck youth up to age 24, and *Circles of Care*, which finds culturally appropriate methods, such as oral traditions, to work with families who have children with severe emotional disturbances. Notably, these programs serve youth and families only; thus, research that specifically focuses on identifying and addressing the complex challenges that Head Start teachers face when working with children and families is necessary. Furthermore, limited information on the cultural strategies used to overcome these challenges to meet programmatic goals and outcomes is available.

## Research Methods

### Community-Based Participatory Research Framework

The decision to use a selected Community-Based Participatory Research (CBPR) framework to guide this study arose from recognizing Native American communities’ distrust toward academic institutions and researchers, which stems from colonization and a history of unethical research practices on their peoples (Brockie et al., 2022). CBPR is the gold standard for research with Native American communities (Anastario et al., 2017; Haroz et al., 2019), as it emphasizes the development of academic-community partnerships, addressing public health concerns identified by the community of interest and centering co-learning as the outcome (Israel et al., 1998). In turn, these actions enhance trust, eliminate power imbalances, and bolster community capacity to reduce health disparities (Wallerstein et al., 2011).

Research team engagement within the community involved: (a) access to guidance from a Native American researcher familiar with the community, (b) attending workshops led by Native American elders and Native academic researchers on how to work with Native populations, and (c) collaboration with other Native and non-Native researchers to conduct a literature review on best practices for culturally safe research with Native American populations (Brockie et al., 2022).

#### Tribal Advisory Board

**Fig. 1:**
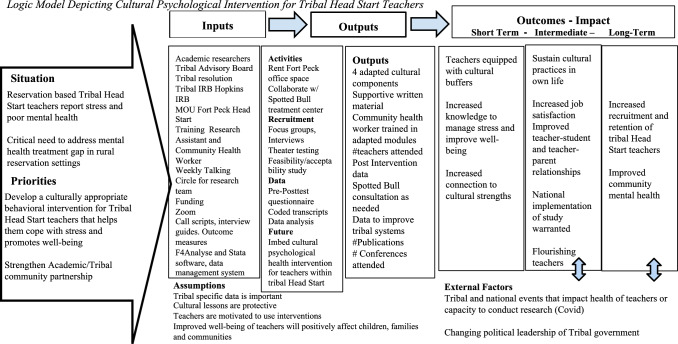
Logic model depicting cultural psychological intervention for tribal head start teachers

In keeping with CBPR, the team established a tribal advisory board comprising of community members who share a common identity, history, language, and culture (Brockie et al., 2022). The board advises researchers on culturally safe research practices and helps their communities understand the research’s rationale, impact, and consent processes (Brockie et al., 2022). Tribal advisory board members receive an honorarium for attending meetings. For this study, the board consisted of a Head Start supervisor, a Head Start teacher, a Head Start parent, a cultural advisor, a public-school educator (and Head Start parent), and a Head Start grandparent. Using an iterative process, we worked with the tribal advisory board to develop a logic model that outlines this study’s research process (see Fig. 1).

### ADAPT-ITT Methodology

Within the CBPR framework, we utilized the ADAPT-ITT methodology to ensure a rigorous process for adapting the *Little Holy One* curriculum. It is also one of the only adaptation models used successfully in Native American contexts and demonstrates a pragmatic focus on adapting evidence-based interventions for culturally diverse populations (Craig Rushing & Gardner, 2016; Wingood & DiClemente, 2008). When applying the ADAPT-ITT process to our study we followed eight sequential steps that guided us through the prescriptive adaptation process:

1. Assessment (literature review, focus groups, interviews).
2. Decision (*Little Holy One*).
3. Adaptation using theater testing methodology.
4. Production (draft of proposed intervention).
5. Topical Experts (tribal advisory board, designers of *Little Holy One*).
6. Integration (final draft that integrates all feedback).
7. Training.
8. Testing.

The ADAPT-ITT designers gave thoughtful consideration in developing a framework attentive to cultural context, sustainability, and targetable for diverse populations—all aspects critically important to our work (Wingood & DiClemente, 2008). For our context, the ADAPT-ITT framework ensured that all aspects of the research process centered on community values, capacity building, and sustainability of the adapted intervention.

**Fig. 2:**
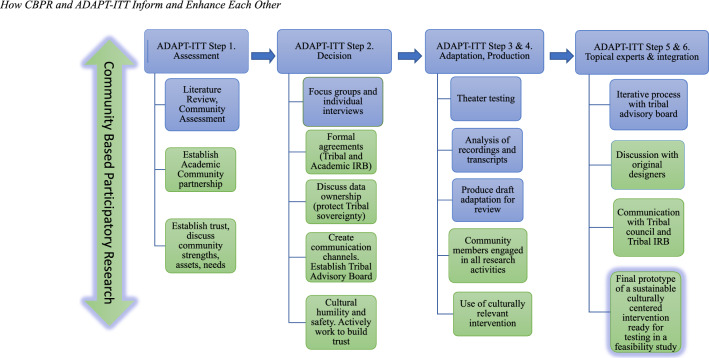
How CBPR and ADAPT-ITT inform and enhance each other

Figure 2 outlines how we employed and intersected our CBPR framework and the first six steps of the ADAPT-ITT methodology for this study. This paper does not report the final two steps of ADAPT-ITT: training and testing. The research team will report on the training of community health workers and the testing of the adapted intervention using a feasibility study in a future publication.

## Data Collection

### Focus Groups and Individual Interviews

We conducted focus groups and interviews with Head Start supervisors, teachers, parents, ancillary staff (e.g., cooks), and community members. These assessed psychological risks such as stress, depression, and PTSD; preferences for intervention content; perceived need(s); and an assessment of capacity within the community to help adapt and adopt the chosen intervention.

Before collecting qualitative data, our tribal advisory board reviewed the semi-structured focus group and in-depth interview guides. They conveyed culturally appropriate ways of ordering the questions and gave feedback on how to ask difficult questions. They also ensured that we took a strengths-based approach to understanding stressors and coping mechanisms among the teachers.

### Recruitment

We conducted recruitment using flyers and email and presented the study purpose at a Head Start staff meeting. A Fort Peck–trained research assistant assessed the interested participants for eligibility. Eligibility criteria included being older than 18; currently working or having worked for Fort Peck Head Start as a teacher, teacher’s assistant, supervisor, or ancillary staff; or being the parents of children who have or had attended Head Start. We offered eligible participants written material about the study and a copy of the consent form to take home for review. The research assistant informed them that participation was entirely voluntary, and worked closely with consenting teachers and parents to schedule convenient interview times. These strategies, and the use of an on-site community member for recruitment, helped to increase retention rates.

Because of the specific thematic focus on teacher stress and coping factors, we used purposive sampling for the interviews and focus groups. Purposive sampling of a targeted population increases the likelihood of information-rich sources, with evidence indicating that such a strategy yields maximum information with fewer sources (van Rijnsoever, 2017).

To ensure a diversity of viewpoints on stressors, well-being, and coping mechanisms within the tribal Head Start workforce, the research team interviewed supervisors, teachers, cultural leaders, council members, and ancillary staff. The team also brought in parents to obtain their perspectives on teacher stress and their needs from teachers, as well as retired teachers and parents whose children have graduated or dropped out of Head Start, to contribute to the diversity and depth of perspective.

Semi-structured interview guides for teacher interviews and focus groups included the following prompts: sources of strengths and stress at work, home, and in the community; describe coping mechanisms; what do you do to promote your well-being; what cultural activities do you employ as part of everyday life or for stress relief; existing support mechanisms and what mechanisms would you like to have to support psychological health. We also asked parents and ancillary staff about their relationships with the teachers; what observations they had about teacher stress; what suggestions they would recommend for teacher support; and what cultural practices they employed for themselves and their children. We added a section to the guides to capture the effect of the COVID-19 pandemic on staff, parents, and the community.

### Interviews and Focus Groups

Due to restrictions on in-person research because of COVID-19, we conducted individual interviews by phone or Zoom. When restrictions eased, our community-based research assistant was able to lead two focus groups, one with teachers (*n* = 5) and one with parents (*n* = 4). Her connection to the community enhanced the trust and ease of participants. One off-site investigator joined on Zoom (as the reservation was closed to non-residents) to take notes and observe.

Before turning on the recorder, we reviewed the consent form to allow for questions and to ensure that everyone understood the nature of the study. We explained that the recordings would be transcribed, deidentified, and used to inform adaptation of the cultural lessons and dissemination activities—eligible teachers who decided not to participate understood that they would not experience any adverse effects on employment. We uploaded signed consent forms into a HIPPA-secure database and destroyed the paper copy to protect participant identity. Participants received a $25 Visa gift card in appreciation for their time. Upon completion of each focus group and interview, the off-site investigator provided a summary of each discussion to secure confirmation or clarification from the participants. This allowed for member checking and increased the trustworthiness of the findings.

We determined theoretical saturation through the observation of repeating themes, and no new information being gathered was reached with a sample size of 27: four administrators/supervisors, seven teachers, four teacher’s assistants, eight parents, and four ancillary staff. Two interview participants chose not to be recorded and were thus interviewed separately, with the facilitator taking notes. We did not include their data in the dissemination activities.

## Data Analysis

We recorded all focus groups and interviews and transcribed them verbatim, and then compared all transcripts to recordings to ensure accuracy. First, we developed a codebook based on the literature and researcher experience volunteering at Head Start. Then using a constructivist approach, two researchers independently coded the qualitative data using the analysis software F4Analyse, meeting weekly to compare coding and discuss emerging new codes and themes. We encouraged diversity of viewpoints and disagreement, to facilitate rich discussion and the emergence of new themes and meta-perspectives (Vaismoradi et al., 2013). Attempts to hire an Indigenous coder were unsuccessful. Thus, while having received training and experience working with indigenous communities, both coders used memos and reflexivity notes while coding and discussed these when meeting with Native American research team members. We explored all emerging themes, questions, and disputes with the Native researchers, one an expert in XXXX (Blinded for review), and one from the *Little Holy One* team, to ensure the inclusion of an indigenous perspective in the thematic analysis. Based on these discussions, themes were either kept, re-coded, or merged.

## Results

Four major themes emerged from the focus groups and interviews: (a) existing mechanisms, (b) mental health and stress, (c) interest in a culturally informed intervention, and (d) sustainability. Quotes are identified as “participant 1–27” to protect participant confidentiality.

### Existing Mechanisms

All participants described existing mental health services as “few” to “non-existent,” with frequent clinician turnover and cultural incongruity identified as significant barriers to peoples’ willingness or ability to attend counseling: “Counselors [who come to the reservation] don’t understand the spirituality of the culture, the poverty, violence, and trauma that took place.” (P2) This lack of understanding and follow-through has led to feelings of “lost promises” (P1) and enhanced reluctance to become involved with supportive care. One participant shared:

“She was on the reservation here, the psychiatrist and stuff, and I worked with her, and I just loved her, and you know how IHS [Indian Health Service] just turns over so many people. They just took her out of here. And someone that you build your trust in and everything, and then they just take her away, and then you got to learn how to talk with someone else and everything, and it’s just not worth it.” (P3)

Teachers, parents, and ancillary staff listed “venting to each other” and “smoking” as available coping mechanism for teachers, with others noting the need for “quiet time at home to wind down.” Teachers described familial support and connections within the culture as primary sources of stress relief: “So, it’s kind of hard to get into mental health or anything like that. I’m just thankful I have my aide and my family that I could talk with and cope with all of this stuff.” (P16) One participant described, “When I lost my husband, I needed support, and I couldn’t get it.” (P18).

### Mental Health and Stress

Stressors varied across all socio-ecological levels. Many teachers mentioned that they care for their grandchildren while maintaining a demanding job: “Yeah, and I’m raising four grandchildren; I’ve had to for six years, and I work.” (P22) All teachers and parents described COVID-19 as a stressor, saying, “COVID kicked us down.” Some said they “can’t even remember being stressed before COVID-19.” Of the 27 participants, 14 described losing a family member or friend to COVID-19, with all describing the toll it took on the community: “I guess the death of a lot of loved ones throughout our community really took a toll.” (P24) “Yeah, it put a black cloud over our reservation.” (P25) One participant also observed, “Teaching doesn’t get the mental health support it needs. Kids commit suicide here, and it affects the teacher.” Another participant pointed out that the teachers might not recognize their stress: “Head Start teachers are always focused on the kids…they don’t know themselves. I think knowing what stress is, what it is, might help.” (P2) Another participant stated that “there is no support for a teacher’s stress.” (P6) Four participants expressed concern that the lack of support for a teacher’s mental health could affect the children: “Teachers are not in a good space, and it doesn’t help the students. The teachers lack knowledge on how to handle stress.” (P8) Another participant acknowledged that “teachers are an important person in a child’s life. They give the child an opportunity to thrive…. They are now in a heightened state of worry [since COVID-19].” (P4).

Eleven participants mentioned the stress of having to deal with special-needs children (e.g., children with autism or children with seizure or emotional disorders): “We see so many more children with behavior issues or autism. Some you can manage in the classroom. Others are totally out of control, yeah.” (P1) Specialized counseling and support for these children is often diverted due to suicides in the community: “We had to make a referral and when it was time for them to come and observe our students and give recommendations...we were having so many suicides with adults or high school kids then, and that always take precedence.” (P1).

A reluctance among Native Peoples to discuss personal issues compounds the lack of access to specialized support: “The way you are raised in Indian country is to not talk about [stress]. We keep a lot in.” (P13) “We are taught to zip it up as a child, and it carries on into adulthood.” (P1) Every participant in the community identified “the drugs, homeless[ness], the crime and no jobs” as everyday stressors. Several teachers and parents described the grief and trauma related to the fallout from substance abuse. Three participants described the heartbreak of losing a child to a drug overdose and the fear of getting another phone call telling them that another child or grandchild was “lost to drugs.” Three participants described their struggles with alcohol addiction after a trauma and their journey to sobriety. Other teachers described the struggles their students face because of parents dealing with substance misuse: “Even with the little kids, what they see in their homes maybe abuse, maybe the alcoholism or some of the kids not even having food in their home.” (P16) Three teachers described violent interactions when engaging with a parent under the influence of drugs or alcohol. They described the stress and fear for their and the child’s safety. One participant summarized, “Sometimes, on the reservation, it isn’t just one trauma. It’s just layer after layer after layer of different traumas that are happening.” (P2).

### Interest in a Culturally Informed Intervention

Connection to the Assiniboine, Sioux, or other Native American traditions among participants was varied. Some stated that they do not routinely practice traditional customs but shared that they partake in ceremonies for “the preparation, outfits, singing, and music.” Others described a regular practice: “If we go somewhere, we’ll smudge before we go, like, kind of protecting us. So yeah, I guess it kind of does give me somewhat of, like, peace of mind.” (P6) Participants, even those who verbalized that they don’t practice the traditional ways, expressed interest in a culturally informed intervention. Notable feedback included “Cultural activities with the adults would be creating that positive [resolving stress, promoting well-being]. Everybody respects the traditional ways and shares an understanding of traditional ways” (P3) and “The suicides, drug abuse, these disparities are related to our loss of culture and language. The more you know about your culture and connect to your community, there is strength in that.” (P16) The following quote powerfully captures the importance of reconnecting to Native culture:

“…. Don’t be ashamed if you don’t know these ways, our cultural ways, or our language. I think there’s a little piece in everyone, especially in the Native community here, that they want to learn that. They want to learn their cultural ways. They want to learn their language and want to have a connection in a sense with their ancestors and their family lineage, you know? There is somebody in their family tree, every person here, that was a cultural person, that did know their language, that did follow these ways. And I think there’s something deep down inside every single person here, I believe, that craves that.” (P8)

Barriers to a culturally informed intervention centered around the strong influence of the religions established by missionaries on the reservation: “There are different religions on the rez, so you may run into some issues [with a cultural intervention].” (P3) “They have been trying to wipe us out [reference to historical genocide]. We need a breakthrough, and we all have a little bit of spirituality in us.” (P18).

### Sustainability

Shared concerns about an intervention’s sustainability included utilizing online services: “Technology is an issue—85% of our teachers don’t know how to use Zoom.” (P1) Additionally, some noted difficulty or reluctance to travel to obtain services: “The wellness center, you know, it would be nice to go there when we are stressed, but it’s hard to get over there. There is nothing else.” (P22).

Administrative staff expressed a willingness to incorporate a culturally informed psychological health intervention within the Head Start framework: “We could incorporate a mental health program into our monthly teacher days on Fridays.” (P2) “The Tribal Handbook pays for 30 min a day for physical health—time to go for a walk, but we have not thought about using it for mental health.” (P1).

In summary, participant input helped identify existing resources, gaps in support, and areas for opportunity with Head Start teachers. It also ascertained the administration’s willingness to work at integrating and sustaining an intervention if deemed feasible and acceptable.

## Suggested Intervention Adaptations

Step three of the ADAPT-ITT process involved theater testing, a pre-testing methodology that presents an original product to an audience aligned with the intended target audience (Wingood & DiClemente, 2008). The research team invited the tribal advisory board, teachers, and community members to a presentation of the four cultural lessons taken from *Little Holy One.* However, at the time of data collection, the Fort Peck Reservation experienced a surge in COVID-19 cases. Thus, participants were limited to the tribal advisory board (five in total), one community-based research assistant, and one off-site investigator to comply with tribal government restrictions on how many people could gather.

We conducted four theater testing sessions over two days. A community research assistant trained to deliver the cultural lessons as intended for *Little Holy One* implemented the modules for this target audience due to her expertise with the original format and knowledge of local tribal culture. Composed of the population to be served, theater session audiences provided critical assessment of the cultural lessons and offered direction for the study team to ensure that the adaptation would meet their needs. This can help to enhance the efficacy of the proposed intervention. The audience could respond and ask questions, allowing the study team to gauge their reaction to the product. The facilitator introduced pertinent data from the qualitative interviews and focus groups, such as “teachers often do not realize the degree of impact of their stress”; “they will not travel to get mental health support”; and “the need for support mechanisms to be made convenient.”

One research team member facilitated a discussion between the presentation of each lesson to elicit feedback and how to adapt to the Head Start teacher context using the following prompts: (a) appropriateness of the lessons for teachers, (b) order in which to implement lessons, (c) additional materials or activities that might enhance its relevance, (d) appropriateness of reflection and activity handouts and assignments, and (e) burden of the intervention.

### Teacher Lessons

Overall, the participants were pleased with the use of traditional cultural practices as a preventative intervention. They felt this could contribute to well-being, tribal self-determination, and reclamation of culture. One participant commented that this was better than seeing an evidence-based Western practice adapted to their tribal context. All participants felt such an intervention would work well, with little adaptation for the teachers. However, some participants also cautioned that some teachers are very Christian and might not feel comfortable immersed in traditional ways. They highlighted the importance of using a community member to teach the cultural lessons, as they would be familiar with interpersonal and community dynamics and the issues of religious versus traditional beliefs. One participant stated very directly “Indians are tired of White people telling them about their traditions. If you have someone from the community implementing the intervention there will be more trust and open discussion. But one of the researchers should implement the stress lesson [managing your emotions] as they are the expert on that.” (Tribal advisory board member).

### Order of Lessons

We discussed changing the delivery order over concerns that the cultural lessons were no longer buffered by the other eight lessons from the original 12-lesson *Little Holy One* RCT. The tribal advisory board suggested placing Smudging before Healing Historical Trauma so that it could be used throughout delivery to deal with any stress that might come up during the discussion of difficult topics. They chose Strengthening Family and Community as the last lesson because it would end the study on a positive note. For the Tribal Identity lesson, tribal advisory board members were concerned that the teachers might feel shame at not knowing their Native language or be shy about trying the greeting activity. They suggested creating an Mp3 with the language examples so that participants could practice at home with their families. Language can be intimidating, they said, and they did not want to make the teachers apprehensive due to fear of mispronunciation. Finally, to decrease the burden of lesson attendance, the tribal advisory board suggested conducting lessons before or after the monthly staff meetings.

### Materials and Activities Lessons

One member asked if the cultural lessons would deal directly with stress. The community research assistant proposed adding a lesson from the other *Little Holy One* eight lessons called “Managing Your Emotions,” which comes from the Common Elements Treatment Approach (CETA) model. The community research assistant presented this lesson to the tribal advisory board, who agreed it would be good to include in the adaption, which increased the proposed adapted curriculum from four to five sessions. The final order was: Tribal Identity; Smudging; Healing Historical Trauma; Managing Your Emotions; and Strengthening Family and Community. *Little Holy One* chose lessons from CETA because of its success in trauma-affected low-resource communities (Murray et al., 2014).

A participant suggested switching the lesson delivery from one-on-one to a group format. This suggestion was well received. The activity handouts would work for a group-based setting, as they involve activities such as building a family tree, which may yield new connections for participants. There were suggestions to add specific group activities, such as preparing a Native meal together or engaging in a traditional sewing activity.

## Preparing the Prototype

After listening to the theater testing session-recordings and reading transcripts, reviewing qualitative themes and field notes, we worked to produce a first draft of the adapted intervention that integrated all comments and feedback (step 5). During production meetings we listed the main outcome of our proposed intervention: to decrease stress and improve well-being using culture as treatment, and went through each lesson, its activities, and handouts to make sure that each aligned with these outcomes and fitted with the new target population of teachers. Importantly we made sure that the elements or activities of each lesson deemed by the original designers as critical to the effectiveness of the intervention were maintained. Examples of production decisions include: (a) children’s activities were removed from each lesson as they are no longer the target, (b) for the Tribal Identity lesson, creation stories were kept in as we agreed that they are an important part of the culture for all age groups, (c) to augment the “greeting one another in Dakota and Nakoda” activity, MP3s of the greetings were made to accompany the handouts to respect potential hesitancy of adults to practice aloud in a group, (d) lesson videos were deemed critical to the core elements of the intervention, (e) for the smudging lesson we decided a participant instead of the interventionist should lead the smudging and added a discussion on if, how and when participants smudge, (f) homework activities were removed due to concerns about participant burden. Each homework activity was assessed, and some were incorporated as part of the lesson e.g., the family tree exercise. By utilizing a careful but rigorous adaptation process informed by community members we hope to enhance intervention fit, improve effectiveness, and maximize fidelity during the upcoming implementation trial.

With a first draft ready we approached our topical experts (Step 6). The original designers of *Little Holy One* expressed concern about the addition of Managing Your Emotions, as it is not a cultural lesson. However, upon review of the qualitative data, in which teachers indicated a lack of awareness or reluctance to talk about their stress, they became amenable to adding content that addressed stress and depression. Their second concern involved the cost and time-intensive nature of the proposed additional activities (preparing a Native meal and sewing), so we discarded these activities. They approved the suggested switch in the order of the lessons. Overall, the original designers felt that the changes did not interfere with the core elements and intent of the cultural lessons and were now appropriate and relevant to the new target population of Head Start teachers.

The research team then took the draft to the tribal advisory board, who went through the adapted five-lesson prototype and listened to the concerns and suggestions made by the *Little Holy One* designers. After reviewing all lessons, the tribal advisory board unanimously approved the prototype for pilot testing in an upcoming feasibility study with Fort Peck Head Start teachers.

The final result was a ready-for-testing five-lesson, culturally informed stress-reduction intervention to promote tribal Head Start teacher well-being and decrease stress and depression. For each lesson, we produced a booklet that included lesson outline, objectives, lesson activities, and suggested follow-up activities. Researchers will hand these booklets out to each participant during intervention implementation. We will report the results from the feasibility study that will assess the implementation of the adapted intervention in a future publication.

## Discussion

The purpose of this paper was to describe the process of adapting a culturally informed intervention to fit tribal Head Start teachers on the Fort Peck Reservation. We designed this study in response to a critical need for psychological and behavioral interventions that are culturally curated for the population(s) they intend to serve and are self-sustaining in rural, low-resource settings. With the lack psychological health support and qualitative data pointing to Head Start teachers being a stressed population in need of structured support, we chose to adapt a preventative psychosocial health intervention that has the potential to provide culturally appropriate psychological support.

Evidence has shown that careful adaptation of interventions can lead to improved engagement and acceptability during the implementation phase, particularly when working with minority populations (Wiltsey Stirman et al., 2019). Using a CBPR framework helped us ensure culturally safe practices and community input throughout, which strengthened the adaptation process. Utilizing the ADAPT-ITT methodology helped provide a rigorous, replicable method so that we can ensure that the intervention will fit with the needs of this specific population.

Our approach yielded necessary adaptions to the original lessons, namely switching from an individual to group format and adding a lesson that explicitly addresses coping with stress and depression. The research team would not have considered these changes without the input of community members and the target population. By reporting on how we worked with the community to adapt this intervention, we hope to contribute to the literature one example of a transparent, systematic process that could help enhance the effectiveness of the upcoming intervention implementation trial. Often the pre-implementation phase is not well documented despite calls for this in the wake of increasing interest in implementation science (Rathod et al., 2018). We hope that such transparency and rigor in reporting the process of this intervention adaptation will allow us to assess the types of modifications that maximize implementation success. The team also paid careful attention to preserving the five lessons’ core elements to ensure fidelity. We feel this will be essential to intervention success with this population.

Our goal was to develop an intervention to improve its feasibility and acceptability with our target population of Head Start teachers and align with their stated priorities, cultural values, and norms. Using interviews and focus groups provided a rich data source for us to gain an understanding of teacher needs, gaps, and existing strengths and supports. Despite being subject to the biases of self-report and being time-consuming to conduct and code, we feel this was critical to adapting an intervention that will be effective for this population.

The research also illuminates the potential efficacy and acceptability of grounding an intervention in traditional cultural practices. Not only is access to effective preventative interventions urgent, but data suggests that psychological treatment-as-usual for Native Americans is often ineffective, as it lacks connection to the culture, they are trying to serve (Gone & Calf Looking, 2011). While still novel, growing evidence exists that immersion in traditional cultural activities promotes healing and well-being (Gone & Calf Looking, 2011; Walls et al., 2016; Wexler, 2013; Wexler & Gone, 2012).

The process outlined in this paper may contribute to the growing evidence of the successes and potential difficulties in adapting psychological health interventions for specific populations and cultural contexts.

### Limitations

This study should be interpreted within the context of its limitations. First, this study is being conducted with a very niche population. We designed the adapted intervention specifically for the Fort Peck Assiniboine and Sioux culture, limiting the sampling frame and generalizability of findings. Second, in-person research was delayed or restricted due to COVID-19. This necessitated that we conduct initial qualitative data collection remotely and for the theater testing sessions to be completed with fewer community members than planned. We would have preferred more significant community input at this data-collection stage; however, the tribal advisory board was representative of the community. The board actively participated in each theater testing session, giving the team the confidence that the adaption process was grounded in community perspectives and needs.

Unexpectedly, the delays in data collection caused by the closure of the reservation due to the rise in COVID-19 cases allowed the research team to develop a working relationship with the Head Start administration. The digital divide was evident, as teachers were unfamiliar with Zoom and thus could not switch to teaching in an online format. Therefore, the research team worked with the administration to assist teachers in developing proficiency with online formats, which allowed an unexpected opportunity for engagement and relationship-building with the target population.

## Conclusion

We thoughtfully designed this study to adapt the four cultural lessons and one CETA lesson from the *Little Holy One* curriculum in preparation for testing in a feasibility study with a sample of tribal Head Start teachers on the Fort Peck Reservation. Community leaders initially developed these cultural lessons to leverage cultural strengths and buffer psychological challenges. Meaningful input from teachers and the tribal advisory board helped ensure that the adapted lessons would apply to the subgroup of teachers and that the cultural lessons’ core values, traditions, and practices remained consistent.

This article presents an example of a replicable process using the principles of CBPR and ADAPT-ITT to allow the conduction of cultural and context-specific adaptations in other settings, with different populations. Adhering to the CBPR framework allowed the prioritization of community perspectives, expertise, and the ongoing recognition of tribal history. Respecting tribal sovereignty and data rights helped develop trust and strengthen the academic-community partnership. The ADAPT-ITT methodology enhanced the relevance and rigor of the adaptation process. Finally, planned future research will explore the feasibility and acceptability of the adapted lessons when piloted with a sample of Fort Peck Head Start teachers. In adapting these lessons for tribal Head Start teachers, we hope to better understand the feasibility and acceptability of providing content centered around their culture. Results may contribute to a greater understanding of developing strengths-based, cultural interventions for educators to decrease stress and enhance their resilience and holistic well-being.

## Supplementary Information

Below is the link to the electronic supplementary material.

Supplementary file1 (DOCX 30 kb)

Supplementary file2 (DOCX 32 kb)

## Data Availability

The government-to-government relationship between the U.S. and federally recognized tribes acknowledges tribal sovereignty, tribal laws, and the tribes’ capacity to make decisions about research that is conducted within their jurisdictional boundaries. The tribal law that supports this research acknowledges the importance of community consent as well as individual consent and will provide the particularities for data sharing as part of tribal research regulation. All resources and data generated by this project will be made available in the most user-friendly, accessible, secure, and ethical format to all study partners. We will take necessary steps to ensure we adhere to Fort Peck Tribal Law (Resolution #30-348-2020-03) and National Institutes of Health guidelines on sharing of data, in collaboration with our tribal partners, including seeking the appropriate tribal approvals to respect their tribal sovereignty and confidentiality. The datasets used and/or analyzed during the current study are available from the corresponding author on reasonable request.

## Abbreviations

ACES: Adverse childhood experiences
CBPR: Community-based participatory research
CETA: Common elements treatment approach
IRB: Institutional Review Board
PTSD: Post-traumatic stress disorder
RCT: Randomized control trial
U.S.: United States
