# Supplementary material for: The Pre-implementation Process of Adapting a Culturally Informed Stress Reduction Intervention for Native American Head Start Teachers
Source: Glob Implement Res Appl. 2023 Jan 9;3(1):16–30. doi: 10.1007/s43477-022-00070-3 (PMC9827016; doi:10.1007/s43477-022-00070-3)
Supplement: Supplementary file 1 — Supplementary file1 (DOCX 30 kb) [file 43477_2022_70_MOESM1_ESM.docx]

| **Item** | **Response** | **Page** |
| --- | --- | --- |
| **Domain 1: Research Team and Reflexivity** | | |
| *Personal Characteristics* | | |
| 1. Interviewer/facilitator | Adriann Ricker led focus group discussions and Deborah Wilson conducted individual interviews. Tribal Advisory Board meetings for guidance on appropriate methods of working within the community and the intervention adaptation were led by Deborah Wilson. Teresa Brockie oversaw the process as Native American advisor. | 13,16,25 |
| 2. Credentials | Teresa Brockie, PhD, MSN, RN – enrolled member of Aaniiian Nation Deborah Wilson MPH, MSN, RN Adriann Ricker, MPH – enrolled member of Assinoboine and Sioux nations  Lydia Sue Koh Krienke MSN, RN | N/A |
| 3. Occupation | Teresa Brockie, Assistant Professor  Deborah Wilson PhD candidate, research associate, RN Adriann Ricker, Research associate, Community liaison and interventionist.  Lydia Sue Koh Krienke research associate, qualitative coding expert | N/A |
| 4. Gender | All researchers are female | N/A |
| 5. Experience and training | Teresa Brockie has worked with Fort Peck Tribes since 2010 and has expertise in suicide and trauma research in Native American populations.  Adriann Ricker is a member of the Fort Peck Tribes and has experience with the design, coding and analysis of qualitative research on the Fort Peck Reservation on a variety of public health topics. Deborah Wilson received formal qualitative research training in her master's degree and PhD program and has expertise with design, coding and analysis of qualitative research. Ms. Wilson also completed training on working with Native American populations and is conducting her dissertation research on the Fort Peck Reservation.  Lydia Koh Krienke completed training on working with Native American populations and has worked as a research assistant on numerous qualitative studies All researchers have qualitative research experience and/or training. | N/A |
| *Relationship with participants* | | |
| 6. Relationship established | Teresa Brockie established a research partnership with Fort Peck Tribes in 2010 and has continued to work in partnership since that time. Deborah Wilson received Tribal IRB approval, Tribal council resolution also obtained for this research and teaches yoga to the Tribal Head Start teachers. Adriann Ricker is a member Fort Peck Tribes and is an interventionist for the *Little Holy* *One* RCT. | 9,13,14 |
| 7. Participant knowledge of the interviewer | Prior to the start of data collection activities participants were presented with an overview of the project. Some participants had also attended a presentation on the *Little* *Holy One* intervention from program staff. Adriann Ricker resides on the Fort Peck Reservation and is a member of the Fort Peck Tribes. | 10,11,13,14,26 |
| 8. Interviewer characteristics | Deborah Wilson and Adriann Ricker have existing relationships and research experiences with Fort Peck Tribes. All researchers are familiar with the *Little Holy* *One* program. | N/A |
| **Domain 2: Study Design** | | |
| *Theoretical Framework* | | |
| 9. Methodological orientation and Theory | Community Based Participatory Research Framework was used to ensure culturally safe research.  Thematic analysis of qualitative interviews during coding  ADAPT_ITT methodology was utilized to guide adaptation process. | 11-,13  21-24 |
| *Participant Selection* | | |
| 10. Sampling | Purposive sampling was utilized. | 14 |
| 11. Method of approach | Tribal Advisory Board (TAB) members were approached for participation via email. Deborah Wilson recruited participants via email and at daylong staff events at Head Start. Adriann Ricker approached additional focus groups participants by phone and email and flyers. | 12,13-15 |
| 12. Sample size | 27 participants joined the study. | 15 |
| 13. Non-participation | Of those that agreed to be part of the study no-one left the interviews, and none refused to participate. 2 refused to be recorded | 15 |
| *Setting* | | |
| 14. Setting of data collection | Interviews were conducted via zoom or over the phone due to COVID-19 restrictions. Focus groups where held in person at the Fort Peck Community College in Poplar, MT. | 14-,17 |
| 15. Presence of non-participants | No. | N/A |
| 16. Description of sample | In order to be included in the study participants needed to currently or previously work for Fort Peck Head Start; or have knowledge of Assiniboine and Sioux culture; or be parents of children that have or had attended Head Start. Participant demographic information was not collected. | 14 |
| *Data Collection* | | |
| 17. Interview guide | Interview guides are available upon request. These interview and focus groups guides were pilot tested on the Tribal Advisory Board | 14-16 |
| 18. Repeat interviews | Two focus groups were conducted, using the same interview guide. No participants completed more than one FGD or interview. | N/A |
| 19. Audio/visual recording | All data collection activities were audio recorded and transcribed. | 1516 |
| 20. Field notes | Field notes were taken during and after the FGDs and all TAB discussions by Deborah Wilson. | 16 |
| 21. Duration | FGDs were approximately 1 1/2 hour long. Individual interviews lasted 40 – 75 mins. | N/A |
| 22. Data saturation | Reached after 27 interviews | 16 |
| 23. Transcripts returned | Transcripts were not reviewed by participants. Transcripts were reviewed by study team members who had participated in data collection activities. A summary of findings was discussed with the TAB. | 17 |
| **Domain 3: Analysis and Findings** | | |
| *Data Analysis* | | |
| 24. Number of data coders | Two researchers coded data separately, and then met to review discrepancies and come to consensus on final codes. | 16 |
| 25. Description of the coding tree | Transcripts were coded using deductive and inductive methods within constructivist framework | 16 |
| 26. Derivation of themes | Themes were identified in advance using a literature search and then some were added inductively during coding | 17-22 |
| 27. Software | F4Analyse | 16 |
| 28. Participant checking | The TAB provided feedback on the findings described and as the represented target group and some had been interviewed it was considered member checking. | 21-23 |
| *Reporting* | | |
| 29. Quotations presented | Yes, participant quotations are included. Focus Group and interview number identifies participant quotes | 17-21 |
| 30. Data and findings consistent | Yes, data noted in results was consistent with overall findings noted in conclusion. | 25-27 |
| 31. Clarity of major themes | Major themes were discussed in the discussion and conclusion. | 26-29 |
| 32. Clarity of minor themes | Not discussed. | N/A |
