# Supplementary material for: The Pre-implementation Process of Adapting a Culturally Informed Stress Reduction Intervention for Native American Head Start Teachers
Source: Glob Implement Res Appl. 2023 Jan 9;3(1):16–30. doi: 10.1007/s43477-022-00070-3 (PMC9827016; doi:10.1007/s43477-022-00070-3)
Supplement: Supplementary file 2 — Supplementary file2 (DOCX 32 kb) [file 43477_2022_70_MOESM2_ESM.docx]

**
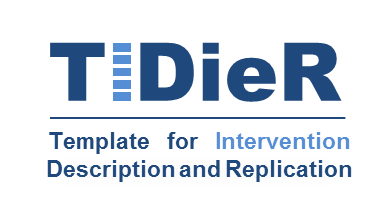
The TIDieR (Template for Intervention Description and Replication) Checklist*:**

Information to include when describing an intervention and the location of the information

| **Item number** | **Item** | **Where located **** | |
| --- | --- | --- | --- |
|  |  | Primary paper  (page or appendix  number) | Other ^†^ (details) |
|  | **BRIEF NAME** |  |  |
| **1.** | **The pre-implementation process of adapting a culturally informed stress reduction intervention for Native American Head Start Teachers** | ______1______ | ______________ |
|  | **WHY** |  |  |
| **2.** | Community Based Participatory Research frames the study to ensure culturally safe research practices with Native American reservation-based participants. ADAPT-ITT methodology provided a prescriptive series of 8 steps to guide the researchers through the adaptation process. | 11,12,13_____ | _____________ |
|  | **WHAT** |  |  |
| **3.** | Materials: Semi structured interview guides (available upon request); The cultural lessons are intellectual property of the tribe and written permission must be made to the Tribal IRB | __13-16_____ | _____________ |
| **4.** | Procedures: Focus groups, individual interviews, Tribal Advisory Board meetings, Theatre testing | 15,16,17-25___ | _____________ |
|  | **WHO PROVIDED** |  |  |
| **5.** | For each category of intervention provider (e.g. psychologist, nursing assistant), describe their expertise, background and any specific training given.  Deborah Wilson MPH, MSN, RN conducted interviews, assisted in focus groups and led theatre testing. Adriann Ricker MPH conducted focus groups and presented the cultural lessons to the Tribal Advisory Board during the Theatre testing exercise. Deborah Wilson received formal qualitative research training in her master's degree and PhD program and has expertise with design, coding and analysis of qualitative research. Ms. Wilson also completed training on working with Native American populations and is conducting her dissertation research on the Fort Peck Reservation.    Both are experience in qualitative interview methods, have completed all human subjects training and Adriann Ricker is an enrolled member of the tribe and resides on the reservation. Adriann Ricker is a member of the Fort Peck Tribes and has experience with the design, coding and analysis of qualitative research on the Fort Peck Reservation on a variety of public health topics. | __N/A_______ | _____________ |
|  | **HOW** |  |  |
| **6.** | Interviews were face-to-face or by phone or zoom. Focus groups were in-person. Theatre testing was in-person. Tribal Advisory Board meetings were either via zoom or in person | _15,16,23,___ | _____________ |
|  | **WHERE** |  |  |
| **7.** | Any in-person activities occurred on the reservation in a hall or conference room at the local community college | _N/A_________ | _____________ |
|  | **WHEN and HOW MUCH** |  |  |
| **8.** | There were 4 theater testing sessions to cover all the original material. The intervention has not yet been implemented. | _21,22_________ | _____________ |
|  | **TAILORING** |  |  |
| **9.** | This whole paper describes the process of adapting the original intervention thus pre-implementation. | _____________ | _____________ |
|  | **MODIFICATIONS** |  |  |
| **10.^ǂ^** | Changed from individual format to group format. Order of lessons changed, and a 5^th^ lesson added. | 22, 23, 24,_25________ | _____________ |
|  | **HOW WELL** |  |  |
| **11.** | Planned: intervention adherence or fidelity will be assessed when the intervention is implemented and will be reported in a future publication. During theatre testing the CHW was assessed for fidelity to original intervention by lead PI | _N/A________ | _____________ |
| **12.^ǂ^** | NA | _____________ | _____________ |

** **Authors** - use N/A if an item is not applicable for the intervention being described. **Reviewers** – use ‘?’ if information about the element is not reported/not sufficiently reported.

† If the information is not provided in the primary paper, give details of where this information is available. This may include locations such as a published protocol or other published papers (provide citation details) or a website (provide the URL).

ǂ If completing the TIDieR checklist for a protocol, these items are not relevant to the protocol and cannot be described until the study is complete.

* We strongly recommend using this checklist in conjunction with the TIDieR guide (see *BMJ* 2014;348:g1687) which contains an explanation and elaboration for each item.

* The focus of TIDieR is on reporting details of the intervention elements (and where relevant, comparison elements) of a study. Other elements and methodological features of studies are covered by other reporting statements and checklists and have not been duplicated as part of the TIDieR checklist. When a **randomised trial** is being reported, the TIDieR checklist should be used in conjunction with the CONSORT statement (see [www.consort-statement.org](http://www.consort-statement.org)) as an extension of **Item 5 of the CONSORT 2010 Statement.** When a **clinical trial** **protocol** is being reported, the TIDieR checklist should be used in conjunction with the SPIRIT statement as an extension of **Item 11 of the SPIRIT 2013 Statement** (see [www.spirit-statement.org](http://www.spirit-statement.org)). For alternate study designs, TIDieR can be used in conjunction with the appropriate checklist for that study design (see [www.equator-network.org](http://www.equator-network.org)).
